# Oral Health-Related Quality of Life throughout Treatment with Clear Aligners in Comparison to Conventional Metal Fixed Orthodontic Appliances: A Systematic Review

**DOI:** 10.3390/ijerph20043537

**Published:** 2023-02-17

**Authors:** Eleftherios G. Kaklamanos, Miltiadis A. Makrygiannakis, Athanasios E. Athanasiou

**Affiliations:** 1 School of Dentistry, Faculty of Health Sciences, Aristotle University of Thessaloniki, 54124 Thessaloniki, Greece; 2 School of Dentistry, European University Cyprus, Nicosia 2404, Cyprus; 3 Hamdan bin Mohammed College of Dental Medicine, Mohammed Bin Rashid University of Medicine and Health Sciences, Dubai 505055, United Arab Emirates; 4 School of Dentistry, National and Kapodistrian University of Athens, 11527 Athens, Greece

**Keywords:** orthodontics, clear aligners, quality of life, OHRQoL, systematic review

## Abstract

Background: Orthodontic clear aligners constitute an alternative and increasingly used orthodontic treatment modality, offering enhanced esthetics with potential consequences regarding patients’ oral health-related quality of life (OHRQoL). Objective: Summarize and systematically evaluate existing evidence on the OHRQoL of patients treated with orthodontic clear aligners compared to treatment with conventional metal, fixed appliances. Search methods: We searched without limitations six databases and searched manually the reference lists of relevant studies up to the end of October 2022. Selection criteria: We looked for data from prospective studies that compared OHRQoL, using instruments that had undergone full psychometric validation, between orthodontic patients with clear aligners and labial, fixed, metal orthodontic appliances. Data collection and analysis: We extracted the data from the located studies, and we assessed the risk of bias with the Cochrane Collaboration suggested tools. The quality of available evidence was based on the GRADE approach. Results: Three studies were identified. OHRQoL was impacted less by clear aligners compared to treatment with conventional labially placed, fixed, metal appliances. The exploratory meta-regression, with the time point of assessment as predictor, did not reveal any statistically significant effect. The quality of the available evidence ranged from very low to low. Conclusions: According to the exploratory synthesis of the limited available dataset, treatment with clear aligners could be associated with better OHRQoL ratings compared to treatment with conventional labially placed, metal, fixed appliances. However, the quality of the presented evidence renders further high-quality studies warranted to be able to reach safer conclusions.

## 1. Introduction

Nowadays, the need for esthetic orthodontic appliances has shown a surge due to the increasing portion of adult orthodontic patients [1]. The classic metal or ceramic brackets are not acceptable by every patient [2,3], and the demand for less visible orthodontic appliances as an alternative to conventional fixed appliances has increased [4,5,6,7]. Regardless of the type of appliance, the aim of orthodontic treatment is to help achieve a healthy, functional and esthetic occlusion, combined with a harmonious facial appearance, which will remain relatively stable in the long run [8]. However, apart from the final occlusal and esthetic result, clinicians should also be interested in the patient-centered impacts of orthodontic treatment and the potential associated health-related implications [9,10].

The concept of quality of life (QoL) is defined as “an individual’s perception of their position in life in the context of the culture and value systems in which they live and in relation to their goals, expectations, standards and concerns” [11]. The parameters of QoL regarding the way people perceive their oral health condition correspond to oral health-related quality of life (OHRQoL) [12]. Overall, an amelioration in OHRQoL following orthodontic treatment has been reported [13,14,15,16], and positive experiences have been noted in social media analyses [17,18]. However, negative impacts on OHRQoL have also been detected, related mainly to pain and functional limitations, as well as concerns related to the appearance of conventional orthodontic appliances [13,14].

A recent systematic review investigated the effect of clear aligner treatment on OHRQoL [19]. However, it retrieved two studies, in one of which the quality of life was assessed at the very beginning of treatment. Furthermore, not all the questionnaires that were used had been psychometrically validated.

## 2. Objectives

To summarize and systematically evaluate existing evidence on the OHRQoL of patients treated with orthodontic clear aligners, compared to treatment with conventional metal, fixed appliances. 

## 3. Materials and Methods

### 3.1. Protocol, Registration and Eligibility Criteria

Pertinent methodological guidelines were followed to form the review protocol [20,21,22,23,24]. We did not seek ethical approval due to the nature of the study.

The PICOS acronym domains determined the eligibility criteria (Supplementary Table S1). The search focused on randomized and non-randomized prospective interventional studies comparing the OHRQoL throughout orthodontic treatment between patients, of any age and gender, with orthodontic clear aligners and conventional metal, fixed, labial appliances. Studies should assess OHRQoL using instruments that had undergone full psychometric validation, including information on test development, validity, reliability and reproducibility [25]. Retrospective studies were not taken into consideration since there could have been significant biases that may have affected the selection of the sample, and because the participants needed to remember what they experienced and how they felt during their treatment.

### 3.2. Information Sources and Search Strategy 

The search was conducted in six databases (Medline (PubMed), CENTRAL (Cochrane Library; includes records from Embase, CINAHL, ClinicalTrials.gov, WHO’s ICTRP, KoreaMed and Cochrane Review Groups’ Specialized Registers, and records identified by handsearching), Cochrane Database of Systematic Reviews (Cochrane Library), Scopus, Web of Knowledge (including Web of Science Core Collection, KCI Korean Journal Database, Russian Science Citation Index, SciELO Citation Index and Zoological Record) and ProQuest Dissertation and Theses (ProQuest)) on 30 October 2022 and was developed by EGK (Supplementary Table S2). Language or date of publication limitations were not imposed. 

### 3.3. Selection Process, Data Collection and Data Items

Retrieved records and the full texts, in the case of unclear abstracts, were evaluated by two authors (EGK and MAM) independently, and pertinent information was documented in predetermined forms. If further clarifications were needed in regard to the published data, or additional materials were necessary, we tried to contact the corresponding authors via email.

### 3.4. Risk of Bias in Individual Studies

Two authors (AEA and MAM) assessed the risk of bias in individual studies independently with ROBINS-I for non-randomized and with the RoB2 tool for RCTs [26,27]. Assessments were visualized using the robvis web application [28]. In all the above-mentioned processes, disagreements were settled by discussion with EGK; following the relevant suggestions, kappa statistics were not calculated [24]. 

### 3.5. Effect Measures and Synthesis Methods

Data on total OHRQoL ratings were reported as standardized mean differences and 95% Confidence Intervals (CI), because the method of calculation of the aggregate score varied across studies. The data at each point of observation were pooled on an exploratory basis with the random effects method for meta-analysis since they were expected to differ across studies due to clinical and methodological diversity. In order to further facilitate the interpretation of the total OHRQoL ratings difference, the standardized mean differences (SMDs) were re-expressed into the initial scale of OHIP-14, based on the information from Jaber et al. [29], and compared to the Minimal Important Difference (MID) in OHRQoL after fixed orthodontic treatment reported by Lau et al. (2022) [30]. Due to the low number of retrieved studies, we did not calculate the corresponding 95% Prediction Intervals [24]. The overlap of the 95% CI was observed graphically, and we calculated the I^2^ statistic [24]. All analyses were performed with Comprehensive Meta-Analysis software version 3.3.070 (©2014 Biostat Inc., Englewood, NJ, USA) (a = 0.05 and 0.10 for the heterogeneity tests [31]).

### 3.6. Certainty Assessment and Additional Analyses

We did not perform the subgroup analyses or analyses for “small-study effects” and publication bias included in the protocol due to the lack of adequate data [24]. Meta-regression was used to explore whether OHRQoL ratings varied by the time point of observation. The quality of evidence for OHRQoL ratings after 1 and 6 months into treatment was evaluated based on the GRADE approach by Guyatt et al. [32], for the purpose of structured and transparent interpretation of the evidence formulation, although extensive information did not exist.

## 4. Results

### 4.1. Study Selection

**Figure 1:**
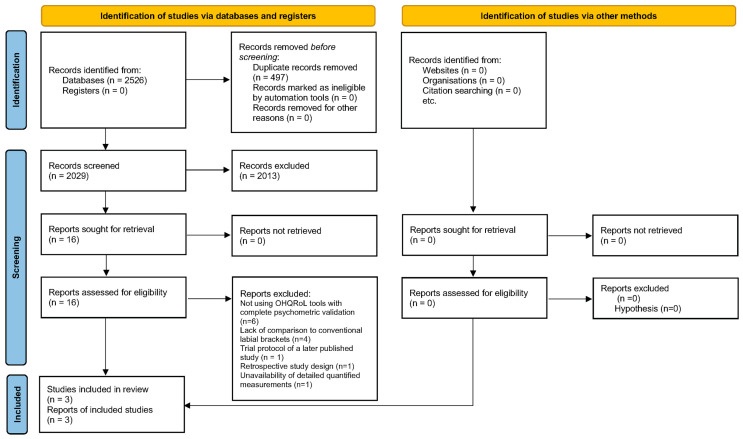
Flowchart of records.

The flow of records is depicted in Figure 1. In total, 2526 records were initially identified and eliminated, 497 as duplicates and 2013 more based on their title and abstract. Out of the 16 records that were kept and assessed for eligibility, 13 records were eliminated for the following reasons: not using a OHRQoL instrument with complete psychometric validation (6); lack of comparison to conventional labial brackets (4); trial protocol of a later published study (1); retrospective study (1); and unavailability of quantified measurements with measures of central tendency and dispersion (1) (Supplementary Table S3). Finally, three studies were included in the systematic review [29,33,34].

### 4.2. Information Sources and Search Strategy

The searches were conducted in six databases on 30 October 2022 and were developed by EGK (Supplementary Table S2). Language or date of publication limitations were not imposed.

### 4.3. Study Characteristics

The characteristics of the eligible studies (published between 2020 and 2022) can be found in Table 1. Two controlled clinical trials [33,34] and one randomized clinical trial [29] were identified, which assessed OHRQoL in a total of 156 patients. Patients were followed, depending on the study, from baseline to the completion of treatment. The clear aligner patients were being treated with either in-house fabricated appliances [29] or using the Invisalign^®^ technique [33], while in the third study, no details were provided [34]. OHRQoL was assessed using the Oral Health Impact Profile-14 (OHIP-14), which is an extensively used instrument with complete psychometric validation. All studies reported the sample size calculation.

### 4.4. Risk of Bias within Studies

**Figure 2:**
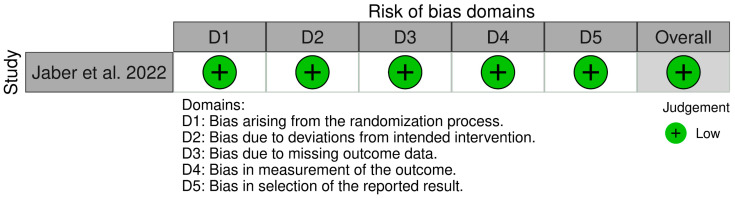
Risk of bias assessment [Randomized Controlled Trials] [29].

**Figure 3:**
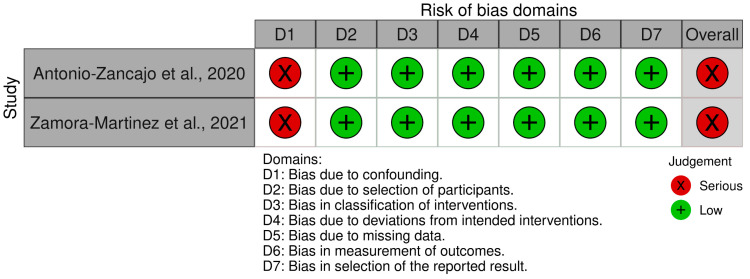
Risk of bias assessment [Controlled Clinical Trials] [33,34].

Figure 2 and Figure 3 present the findings of the risk of bias assessment for the randomized clinical trial and the two controlled clinical trials, respectively. Jaber et al. [29] was assessed to be generally at low risk of bias. The studies by Antonio-Zancajo et al. [33] and Zamora-Martinez et al. [34] were detected to be at a serious risk of bias due to confounding, as important domains, such as socioeconomic status and age variation [35,36], were not measured in an appropriate way or were not controlled for. Both are factors related to the patients themselves. However, both can affect the way participants perceive their OHRQoL.

### 4.5. Oral Health-Related Quality of Life during Treatment

**Figure 4:**
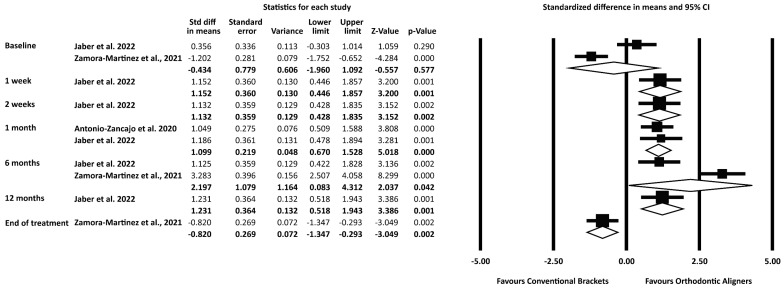
Forest Plot of the oral health-related quality of life (OHRQoL) ratings in patients treated with orthodontic aligners compared to those treated with conventional labial brackets [29,33,34].

No differences were noted in total OHRQoL ratings between patients treated with clear aligners and conventional labial, metal appliances at the start and the end of treatment. During treatment, patients treated with clear aligners reported fewer impacts (Figure 4) (Supplementary Table S4). The differences corresponded to a 2.5-point difference in the total OHIP-14 scale at 1 week, 2 weeks, 1 month and 12 months into treatment, and they were less than the MID reported by Lau et al. (2022) [30]. However, the difference in the total OHIP-14 scale after 6 months into treatment corresponded to 23 points, more than the MID reported by Lau et al. (2022) [30]. The results of the exploratory meta-regression did not show any statistically significant variation in OHRQoL assessment with time (Table 2). The quality of available evidence for total OHIP-14 ratings after 1 and 6 months into treatment was assessed to be low and very low, respectively (Table 3).

## 5. Discussion

Based on the findings of the exploratory synthesis of OHRQoL data from psychometrically validated instruments, which was presented in the current systematic review, treatment with aligners may bring additional benefits to the reported increases in OHRQoL conferred by orthodontic correction [13,14,15,16], when compared to treatment with conventional labially placed, metal, fixed appliances. Such benefits might reflect on patients’ comfort while sleeping and eating and during social contacts, as well as on a person’s self-esteem and overall satisfaction with oral health [10]. The quality of the presented evidence was not assessed highly, and relevant recommendations should be approached cautiously. However, the clinician should not overlook the possible implications of clear aligner treatment on OHRQoL, when treatment effectiveness is not an issue.

Nowadays, it can no longer be argued that patient-reported outcomes are paramount when addressing the impact from a disease and its treatment, at least in non-life-threatening conditions [37]. However, assessing the perspective of patients might prove to be challenging, since measurement instruments should have demonstrated acceptable essential psychometric properties, such as validity and reliability, if their results are to be considered trustworthy [36]. For this reason, studies not using OHRQoL instruments with complete psychometric validation were excluded from the present review [25], although they present corroborating information.

Miller and co-workers [38] studied the impacts on functional and psychosocial-related domains during the first week of treatment with a daily diary, in adults treated with aligners and conventional fixed appliances. Patients of the first group reported a lower number of negative impacts on the overall QoL, as well as the subscales evaluated, in comparison to the fixed appliances group.

Furthermore, another two prospective studies examined the adjustment of adults over the first two weeks of treatment with labial-metal-fixed and clear aligner orthodontic appliances [39,40]. A sample of 68 individuals reported on oral dysfunction (difficulty speaking, swallowing or opening the mouth), eating disturbances (difficulty eating, reduced pleasure of food and taste changes), general activity parameters and oral symptoms. The group treated with aligners demonstrated the least oral symptoms. The general activity problems and oral dysfunction in the group were similar to the labial appliance patients, and the overall adaptation was uneventful and affected to a small degree by psychological traits, such as grandiosity, obsessive–compulsive characteristics, somatization, depression, hostility, anxiety and paranoid ideation.

Alajmi et al. [41] conducted an observational retrospective study involving 60 adults (30 treated with the Invisalign^®^ technique and another 30 with conventional labial fixed appliances) who filled out a questionnaire evaluating oral impacts and treatment satisfaction. The authors concluded that treatment with orthodontic aligners is not always more pleasant, but it can be easily tolerated, as it fulfils needs regarding food consumption and helps eliminate ulcerations of the mucosa. Nevertheless, clear aligners may influence speech regarding pronunciation and delivery in the short run.

Similar results were found in the study of Baseer et al. [42]. They carried out a cross-sectional observational study on 150 adult patients recruited to complete a self-administered questionnaire, 118 of whom were treated with fixed orthodontic appliances and 32 with the Invisalign^®^ technique. The questionnaire included items on daily routine, the consumption of food and possible oral symptoms 1 week after the activation of the appliances. Patients in the fixed appliance group reported having more pain, difficulties in sleeping, impaction of food and irritations on the tongue and cheeks after the first week of treatment [42].

The exploratory synthesis of OHRQoL data from psychometrically validated instruments suggested statistically significant superior OHRQoL ratings for the clear aligner group at all time points assessed during treatment, which is consistent with the findings of the exploratory meta-regression that did not reveal any statistically significant effect of the time point of OHRQoL assessment. Although the differences for most points did not exceed the Minimally Important Difference for the total OHIP-14 scale as reported by Lau et al. (2022) [30], at 6 months after treatment initiation, the difference was more than the MID and, thus, can be considered to be clinically significant, as well. MID has been defined as “the smallest difference in score in the domain of interest that is considered to be clinically meaningful, which patients perceive as beneficial” [43].

Meanwhile, at the timepoint of the end-of-treatment assessment, a “plot twist” was detected; the group treated with conventional labial metal brackets reported better OHRQoL compared to those treated with clear aligners. This unanticipated, at-first-sight result could be attributed to the imbalance present also at the baseline assessment [34]. It is possible that the lack of assessment or control for important parameters, such as the socioeconomic and age characteristics of the patients, led to confounding effects, as presented also in the risk of bias investigation section.

In the same context, it is worth mentioning that OHRQoL may be affected by factors other than the type of appliance. Age and tooth extractions, combined with orthodontic treatment, socioeconomic factors and other parameters, could affect patient perceptions [35,36]. Some of the included studies included participants with a wide age range, which could have affected the OHRQoL ratings. Jaber et al. [29] studied a patient sample from 18 to 25 years; Antonio-Zancajo et al. [33] included adults up to 40 years of age; and Zamora-Martinez et al. [34] assessed OHRQoL in individuals up to 68 years of age. The Jaber et al. study [29] included only orthodontic patients who had undergone premolar extractions first and whose sociodemographic characteristics were not thoroughly reported. Only one study investigated the latter [34]. From this information, it is obvious that future research should focus on accounting for these confounding factors.

Although the issues of the efficiency and effectiveness of clear aligner orthodontic treatment in comparison to conventional fixed appliances mechanotherapy are not part of this report, it should be noted that there is a discrepancy between the advanced, rapid pace of developments in the field of clear aligner orthodontic therapy and the status of relevant scientific documentation and evidence. Therefore, clear aligner treatments require further and comprehensive documentation regarding their evidence-based outcome perspectives [44].

### 5.1. Strengths and Limitations

The strengths of the present review include using an established methodology and focusing exclusively on comparative studies between patients treated with aligners or metal fixed appliances. The search strategy employed was as exhaustive and comprehensive as possible, covering grey literature, as well as electronic and manually searched, up to the end of October 2022. The heterogeneity ensuing from the clinical and methodological variability was incorporated in the random effects model employed in the exploratory quantitative data synthesis [26]. Limitations emerge because of the low number of included studies and the nature of the studies and samples. Although it is not impossible to address the blinding of the participants regarding the type of appliance used, the authors of the present study do not feel that this methodological deficiency could incur bias in the context of the study design under consideration.

### 5.2. Recommendations for Future Research

Future well-designed studies could focus on the use of psychometrically validated questionnaires and should limit potential confounding factors that could have an impact on OHQRoL.

## 6. Conclusions

Based on the exploratory synthesis of the limited available dataset, treatment with clear aligners could be associated with better OHRQoL ratings compared to treatment with conventional labially placed, metal, fixed appliances. However, the quality of the presented evidence renders further high-quality studies warranted in order to be able to reach safer conclusions.

## Figures and Tables

**Table 1:** Characteristics of studies included in the systematic review.

Studies	Participant Characteristics	Orthodontic Treatment Characteristics and Outcomes Assessed
**Antonio-Zancajo et al. (2020)** **Spain** **CCT** **[33]**	**60 orthodontic patients** [31 F, 29 M] **Criteria [+]** 18–40 y, permanent dentition, no previous extractions, dental bone discrepancy between −2 and −6 mm in both arches, good oral health without caries or periodontal disease, skeletal Class I or mild Class II and 3 (ANB 0–5); **[-]** deciduous teeth or in the process of dental replacement, need for orthodontic surgical treatment or extractions, systemic diseases, medication that influences pain perception, severe malformations, anatomy of the lingual side preventing the cementation of lingual brackets in the lingual group **Sample size calculation:** mentioned	**Conventional brackets:** Victory Series^®^, 3 M; 30 (13 M, 17 F, mean age 24.7 y) **Orthodontic aligners:** Invisalign^®^, Align Technology; 30 (16 M, 14 F, mean age 33.4 y) **Oral Health Impact Profile-14 (OHIP-14)** Assessment: 1 m
**Jaber et al. (2022)** **Syria** **RCT** **[29]**	**36 orthodontic patients** [19 F, 17 M] **Criteria [+]** (1) Age: 18–25 y, (2) Class I malocclusion with severe crowding (more than 5 mm of tooth size-arch length discrepancy) and a score of 25 points and above according on the American Board of Orthodontics Discrepancy Index (ABO-DI), (3) No congenitally missing or extracted teeth (except for the third molars), (4) No history of previous trauma to the maxillofacial region or surgical interventions; **[-]** (1) Previous orthodontic treatment, (2) Patients with psychological abnormalities, (3) Patients with systematic diseases, and (4) Patients who have known allergies to latex and plastic **Sample size calculation:** mentioned	**Conventional brackets:** Master Series^®^, American Orthodontics; 18 (8 M, 10 F, mean age 20.86 y) **Orthodontic aligners:** In house aligners; 18 (9 M, 9 F, mean age 21.27 y) **Oral Health Impact Profile-14 (OHIP-14)** Assessment: baseline, 1 w, 2 w, 1 m, 6 m, 12 m
**Zamora-Martinez et al. (2021)** **Spain** **CCT** **[34]**	**60 orthodontic patients** **Criteria [+]** Age > 18 y, good oral health (without caries or periodontal disease), good general health; **[-]** orthognathic surgery, previous orthodontic treatment, missed more than three appointments, systemic disease. Incomplete protocol due to a lack of patient collaboration: (1) failure to follow the treatment regimen; (2) forms completed incorrectly, unwilling to take part in the study **Sample size calculation**: mentioned	**Conventional brackets:** 30 **Orthodontic aligners:** 30 **Oral Health Impact Profile-14 (OHIP-14)** Assessment: baseline, 6 m, end of treatment

CCT: Controlled Clinical Study; F: female; M: male; m: month; RCT: randomized clinical trial; w: week; y: years; [+]: inclusion criteria; [-]: exclusion criteria.

**Table 2:** Main results and statistics for the regression model.

Main Results ^1^						
Covariate	Coefficient	SE	LL	UL	Z-Value	2-Sided *p*-Value
**Intercept**	1.0118	0.4989	0.034	1.9896	2.03	0.0425
**Time point of OHRQoL assessment (in days)**	−0.0012	0.0019	−0.0048	0.0025	−0.63	0.5288
**Statistics**						
**Test of the model:** Q = 0.40, df = 1, *p* = 0.5288						
**Goodness of fit:** Tau² = 1.5720, Tau = 1.2538, I² = 93.29%, Q = 119.17, df = 8, *p* = 0.0000						
**Total between-study variance:** Tau² = 1.4591, Tau = 1.2079, I² = 93.11%, Q = 130.65, df = 9, *p* = 0.0000						
**Proportion of total between-study variance explained by the model:** R² analog = 0.00						

^1^ Random effects (Method of Moments), Z-Distribution. LL: Lower limit; SE: Standard Error; UL: Upper limit.

**Table 3:** Quality of available evidence.

Quality Assessment						Effect Size	Quality
Studies	Risk of Bias	Inconsistency	Indirectness	Imprecision	Other	SDM and 95% CI	
**Total OHIP-14 ratings after 1 month into treatment**							
2 datasets	Serious	Not serious	Not serious	Serious	None	1.099 more in the Conventional Bracket Group [from 0.670 to 1.528] *p* = 0.000	⊕⊕◯◯ **LOW**
**Total OHIP-14 ratings after 6 months into treatment**							
2 datasets	Serious	Serious ^1^	Not serious	Serious	None	2.197 more in the Conventional Bracket Group [from 0.083 to 4.312] *p* = 0.042	⊕◯◯◯ **VERY LOW**

CI: Confidence Interval; SMD: Standardized difference in means; ^1^ Inconsistency was 94%.

## Data Availability

The data underlying this article derive from those included in the relevant published articles that were included.

## Supplementary Materials

The following supporting information can be downloaded at: https://www.mdpi.com/article/10.3390/ijerph20043537/s1. Table S1. Eligibility criteria. Table S2. Strategy for database search [October 2022]. Table S3. Excluded studies with reasons. Table S4. Q-test and I2 statistic results.
