# Supplementary material for: Oral Health-Related Quality of Life throughout Treatment with Clear Aligners in Comparison to Conventional Metal Fixed Orthodontic Appliances: A Systematic Review
Source: Int J Environ Res Public Health. 2023 Feb 17;20(4):3537. doi: 10.3390/ijerph20043537 (PMC9961780; doi:10.3390/ijerph20043537)
Supplement: Supplementary file 1 [file ijerph-20-03537-s001.zip › ijerph-2204868-supplementary.pdf]

Supplementary Table S1. Eligibility criteria.

Domain	Inclusion criteria	Exclusion criteria
**Participants**	• Individuals of any age and gender under comprehensive orthodontic treatment.	• Patients at an initial phase of removable or functional appliance treatment, patients during combined orthodontic and surgical treatment.<br>• Subjects with craniofacial anomalies or syndromes of the head and neck region, as well as physical or mental handicaps.
**Intervention**	• Orthodontic treatment with orthodontic aligners	
**Comparator**	• Orthodontic treatment with labial fixed metal orthodontic appliances.	
**Outcomes**	• Quantified measurements (mean values together with the respective standard deviations or other measures used to quantify the central tendency and the degree of dispersion of a set of data values) on oral-health quality of life using instruments that have undergone full psychometric validation (Streiner et al., 2014).	• Oral-health quality of life and treatment satisfaction using instruments not fully psychometrically validated (Streiner et al., 2014).
**Study design**	• Experimental prospective controlled studies (according to the Scottish Intercollegiate Guidelines Network algorithm for classifying study design (available at http://www.sign.ac.uk/ assets/study_design.pdf).	• Non-comparative and retrospective studies, reviews, systematic reviews and meta-analyses

Streiner DL, Norman GR, Cairney J. Health Measurement Scales: A practical guide to their development and use (5th Ed.), Oxford University Press, 2014.

Supplementary Table S2. Strategy for database search [October 2022].

Database [2022 10 30]	Search strategy	Hits
**PubMed**	(report*[tiab] OR perspective*[tiab] OR rate*[tiab] OR rati*[tiab] OR “rating scale”[tiab] OR “rating scales”[tiab] OR assessment*[tiab] OR experience*[tiab] OR pain[tiab] OR “quality of life”[tiab] OR “quality-of-life”[tiab] OR qol[tiab] OR satisfaction[tiab] OR wellbeing[tiab] OR “well being”[tiab] OR well-being[tiab]) AND ((Orthodont*[tiab] OR Clear[tiab] OR transparent[tiab] AND aligner*[tiab]) OR Invisalign[tiab])	**365**
**Cochrane Central Register of Controlled Trials**	(report* OR perspective* OR rate* OR rati* OR “rating scale” OR “rating scales” OR assessment* OR experience* OR pain OR “quality of life” OR “quality-of-life” OR qol OR satisfaction OR wellbeing OR “well being” OR well-being) AND ((Orthodont* OR Clear OR transparent AND aligner*) OR Invisalign)	**518**
**Cochrane Database of Systematic Reviews**	(report* OR perspective* OR rate* OR rati* OR “rating scale” OR “rating scales” OR assessment* OR experience* OR pain OR “quality of life” OR “quality-of-life” OR qol OR satisfaction OR wellbeing OR “well being” OR well-being) AND ((Orthodont* OR Clear OR transparent AND aligner*) OR Invisalign)	**2**
**Scopus**	TITLE-ABS (report* OR perspective* OR rate* OR rati* OR "rating scale" OR "rating scales" OR assessment* OR experience* OR pain OR "quality of life" OR "quality-of-life" OR qol OR satisfaction OR wellbeing OR "well being" OR well-being) AND ((orthodont* OR clear OR transparent AND aligner*) OR invisalign) AND (LIMIT-TO (SUBJAREA , "DENT"))	**637**
**Web of Science™**	(report* OR perspective* OR rate* OR rati* OR “rating scale” OR “rating scales” OR assessment* OR experience* OR pain OR “quality of life” OR “quality-of-life” OR qol OR satisfaction OR wellbeing OR “well being” OR well-being) AND ((Orthodont* OR Clear OR transparent AND aligner*) OR Invisalign) (Title) and (report* OR perspective* OR rate* OR rati* OR “rating scale” OR “rating scales” OR assessment* OR experience* OR pain OR “quality of life” OR “quality-of-life” OR qol OR satisfaction OR wellbeing OR “well being” OR well-being) AND ((Orthodont* OR Clear OR transparent AND aligner*) OR Invisalign) (Abstract) and Dentistry Oral Surgery Medicine (Research Areas) and Humans (MeSH Headings)<br>All databases; Timespan: All years. Search language=Auto	**894**
**ProQuest Dissertations and Theses Global**	title((Orthodont* OR Clear OR transparent AND aligner*) OR Invisalign) AND abstract((Orthodont* OR Clear OR transparent AND aligner*) OR Invisalign) [Full text]	**110**

Supplementary Table S3. Excluded studies with reasons.

Excluded studies	Reason
Alajmi S, Shaban A, Al-Azemi R. Comparison of Short-Term Oral Impacts Experienced by Patients Treated with Invisalign or Conventional Fixed Orthodontic Appliances. Med Princ Pract. 2020;29:382-388	OHQRoL tool without complete psychometric validation
Baseer MA, Almayah NA, Alqahtani KM, Alshaye MI, Aldhahri MM. Oral Impacts Experienced by Orthodontic Patients Undergoing Fixed or Removable Appliances Therapy in Saudi Arabia: A Cross-Sectional Study. Patient Prefer Adherence. 2021 Dec 2;15:2683-2691.	OHQRoL tool without complete psychometric validation
Cooper-Kazaz R, Ivgi I, Canetti L, Bachar E, Tsur B, Chaushu S, Shalish M. The impact of personality on adult patients' adjustability to orthodontic appliances. Angle Orthod. 2013 Jan;83(1):76-82	OHQRoL tool without complete psychometric validation
Flores-Mir C, Brandelli J, Pacheco-Pereira C. Patient satisfaction and quality of life status after 2 treatment modalities: Invisalign and conventional fixed appliances. Am J Orthod Dentofacial Orthop. 2018 Nov;154(5):639-644.	Retrospective study design
Gao M, Yan X, Zhao R, Shan Y, Chen Y, Jian F, Long H, Lai W. Comparison of pain perception, anxiety, and impacts on oral health-related quality of life between patients receiving clear aligners and fixed appliances during the initial stage of orthodontic treatment. Eur J Orthod. 2021 Jun 8;43(3):353-359.	Unavailability of detailed quantified measurements
Jaber ST, Hajeer MY. Comparison of Oral Health Related Quality of Life Changes Between Clear Aligners and Vestibular Fixed Appliances. NCT04866238	Protocol of a later published study
Miller KB, McGorray SP, Womack R, Quintero JC, Perelmuter M, Gibson J, Dolan TA, Wheeler TT. A comparison of treatment impacts between Invisalign aligner and fixed appliance therapy during the first week of treatment. Am J Orthod Dentofacial Orthop. 2007 Mar;131(3):302.e1-9.	OHQRoL tool without complete psychometric validation
Nicholson KC. A survey study comparing adult orthodontic patient quality of life between Invisalign and fixed appliances. Thesis, 2011	OHQRoL tool without complete psychometric validation
Pacheco-Pereira C, Brandelli J, Flores-Mir C. Patient satisfaction and quality of life changes after Invisalign treatment. Am J Orthod Dentofacial Orthop. 2018 Jun;153(6):834-841.	Lack of comparison to conventional labial brackets
Sauer MK, Drechsler T, Peron PF, Schmidtmann I, Ohlendorf D, Wehrbein H, Erbe C. Aligner therapy in adolescents: first-year results on the impact of therapy on oral health-related quality of life and oral hygiene. Clin Oral Investig. 2022 Oct 29.	Lack of comparison to conventional labial brackets
Shalish M, Cooper-Kazaz R, Ivgi I, Canetti L, Tsur B, Bachar E, Chaushu S. Adult patients' adjustability to orthodontic appliances. Part I: a comparison between Labial, Lingual, and Invisalign™. Eur J Orthod. 2012 Dec;34(6):724-30	OHQRoL tool without complete psychometric validation
Xu L, Li H, Mei L, Li Y, Wo P, Li Y. Aligner treatment: patient experience and influencing factors Australasian Orthodon J. 2022;38:88-95	Lack of comparison to conventional labial brackets
Xu L, Mei L, Lu R, Li Y, Li H, Li Y. Predicting patient experience of Invisalign treatment: An analysis using artificial neural network. Korean J Orthod. 2022 Jul 25;52(4):268-277.	Lack of comparison to conventional labial brackets

Supplementary Table S4. Q-test and I2 statistic results.

		Heterogeneity			
**Time point**	**Number of studies**	**Q-value**	**df (Q)**	**P-value**	**I-squared**
**[00] Baseline**	2	12.663	1	0.000	92
**[02] 1 week**	1	0.000	0	1.000	0
**[03] 2 weeks**	1	0.000	0	1.000	0
**[04] 1 month**	2	0.091	1	0.762	0
**[05] 6 months**	2	16.329	1	0.000	94
**[06] 12 months**	1	0.000	0	1.000	0
**[07] at the end of Tx**	1	0.000	0	1.000	0
